# A randomized controlled trial of comparative effectiveness between the 2 dose and 3 dose regimens of hepatitis a vaccine in kidney transplant recipients

**DOI:** 10.1038/s41598-020-80052-3

**Published:** 2021-01-08

**Authors:** Thaninee Prasoppokakorn, Jakapat Vanichanan, Roongruedee Chaiteerakij, Kamonwan Jutivorakool, Suwasin Udomkarnjananun, Krit Pongpirul, Wipusit Taesombat, Salin Wattanatorn, Yingyos Avihingsanon, Kriang Tungsanga, Somchai Eiam-Ong, Kearkiat Praditpornsilpa, Natavudh Townamchai

**Affiliations:** 1 Division of Gastroenterology, Department of Medicine, Faculty of Medicine, Chulalongkorn University and King Chulalongkorn Memorial Hospital, Bangkok, Thailand; 2 Division of Infectious Diseases, Department of Medicine, Faculty of Medicine, Chulalongkorn University and King Chulalongkorn Memorial Hospital, Bangkok, Thailand; 3 Center of Excellence for Innovation and Endoscopy in Gastrointestinal Oncology, Faculty of Medicine, Chulalongkorn University, Bangkok, Thailand; 4 Division of Nephrology, Department of Medicine, Faculty of Medicine, Chulalongkorn University and King Chulalongkorn Memorial Hospital, Bangkok, Thailand; 5 Excellence Center for Solid Organ Transplantation, King Chulalongkorn Memorial Hospital, Bangkok, Thailand; 6 Renal Immunology and Renal Transplant Research Unit, Chulalongkorn University, Bangkok, Thailand; 7 Department of Preventive and Social Medicine, Faculty of Medicine, Chulalongkorn University, Bangkok, Thailand; 8 Department of Surgery, Faculty of Medicine, Chulalongkorn University and King Chulalongkorn Memorial Hospital, Bangkok, Thailand

**Keywords:** Immunology, Transplant immunology, Vaccines, Microbiology, Vaccines

## Abstract

Hepatitis A virus (HAV) is able to cause a spectrum of illnesses ranging from no symptom to fulminant hepatitis which may lead to acute kidney injury. Although hepatitis A vaccine is recommended in non-immune solid organ transplant recipients who live in or travel to endemic areas, the standard 2-dose vaccination regimen demonstrated less favorable immunogenicity among these population. The 3-dose regimen showed higher response rate and immune durability in patients with human immunodeficiency virus. However, this strategy has never been studied in solid organ transplant recipients. A single-center, open-labeled, computer-based randomized controlled trial (RCT) with a 2:1 allocation ratio was conducted from August 2017 to December 2018. The study compared the seroconversion rate after receiving 2- or 3-dose regimen of hepatitis A vaccine at 0, 6 and 0, 1, 6 months, respectively, in non-immune kidney transplant recipients. A total of 401 adult kidney transplant recipients were screened for anti-HAV IgG and 285 subjects had positive results so the seroprevalence was 71.1%. Of 116 seronegative recipients, 93 (80.2%) completed vaccination; 60 and 33 participants completed 2- and 3-dose vaccination, respectively. The baseline characteristics were comparable between both groups. The seroconversion rate at 1 month after vaccination was 51.7% in the standard 2-dose regimen and 48.5% in the 3-dose regimen (p = 0.769). Overall, the seroconversion rate appeared to be associated with high estimated glomerular infiltration rate, high serum albumin, and low intensity immunosuppressive regimen. Seroconversion rate after hepatitis A vaccination in kidney transplant recipients was less favorable than healthy population. Three-dose regimen did not show superior benefit over the standard 2-dose regimen. Other strategies of immunization may increase immunogenicity among kidney transplant recipients.

## Introduction

Hepatitis A virus (HAV) is one of the most common etiologies of acute hepatitis worldwide, especially in developing countries. Clinical presentations of HAV infection range from no symptom to fulminant hepatitis^1–3^. Improvement in health status and economic status lead to reduced HAV infection but this can result in decreased immunity to HAV, particularly in subjects younger than 50 years old^4–7^. Therefore, HAV infection is mostly mild and self-limited in children. On the other hand, there was a high risk for HAV infection in adult population and immunocompromised patients, including subjects undergoing solid organ transplantation (SOT). These two populations are more likely to have severe disease, resulting in substantial morbidity and mortality^2,8,9^. Furthermore, acute kidney injury can be found in patients with fulminant hepatic failure^10^. Hence, kidney transplant recipients are at high risk of allograft loss.

Since there are no specific antiviral treatment, thus vaccination against HAV is crucial to prevent HAV infection among at risk population. Hepatitis A vaccine is an inactivated vaccine which is safe and can be used in immunocompromised patients^11^. The standard 2-dose vaccination regimen, administered at 0 and 6 months, is recommended for all individuals who live in or travel to endemic areas regardless of immune status^12^. According to the guidelines, hepatitis A vaccine should be given to either SOT candidates or recipients who have negative anti-HAV immunoglobulin G (IgG)^13,14^. However, there were scarce data on anti-HAV seroprevalence of standard 2-dose regimen in SOT and the results varied between countries^15–17^.

In kidney transplant recipients, the standard 2-dose regimen yielded less favorable immunogenicity and the seroconversion rate was 26.9–71.8% compared to the healthy population which had a seroconversion rate of more than 95%^15,17^. To enhance the percentage of seroconversion, the standard hepatitis A vaccine plus an additional dose, 3-dose regimen, have been evaluated in immunocompromised subjects. In patients with human immunodeficiency virus (HIV), the 3- dose hepatitis A regimen could provide greater immune response rate and longer immune durability compared to the standard 2-dose regimen^18,19^. However, this strategy has never been evaluated in SOT recipients.

This prospective, randomized controlled trial is the first of its kind to assess HAV seroepidemiology and compare the effectiveness of the 2- and 3-dose regimens of hepatitis A vaccination among kidney transplant recipients.

## Methods

### Study design and participants

A single-center, open-labeled, computer-based randomized controlled study was conducted at the King Chulalongkorn Memorial Hospital, Bangkok, Thailand, from August 2017 to December 2018. The inclusion criteria were kidney transplant recipients older than 18 years old who had kidney transplantation for more than 6 months and on standard immunosuppressive regimens with stable allograft function for at least 3 months. The main immunosuppressive regimen consists of tacrolimus, mycophenolic acid (mycophenolate mofetil or enteric-coated mycophenolate sodium) and prednisolone. Some patients, if clinically indicated, were on immunosuppressive minimization regimens which consisted of mammalian target of rapamycin inhibitor (mTOR inhibitor) together with calcineurin inhibitor (CNI) and prednisolone or mTOR inhibitor with mycophenolic acid and prednisolone. Kidney transplant recipients with active rejection or infection within 3 months before screening were excluded from the study. All participants provided written informed consent prior to their enrollment in the study and medical records were thoroughly reviewed. The study was approved by the Institutional Review Board of the Research Ethics Review Committee for Research Involving Human Research Participants, Health Sciences Group, Chulalongkorn University (610/60), and is in accordance with the Helsinki Declaration of 1983. The study was registered in Thai Clinical Trials Registry (TCTR20180325001).

### Procedures

We evaluated seroprevalence of anti-HAV IgG in all consecutive kidney transplant recipients during the study period. The anti-HAV IgG was measured using the automated chemiluminescent microparticle immunoassay on the ARCHITECT i2000SR instrument (ARCHITECT HAVab-IgG, Abbott, Wiesbaden, Germany) according to the manufacturer’s instructions. The sensitivity and specificity of the test were > 98% and > 99.17%, respectively. Samples with the signal-to-cut-off (S/CO) ratio of > 1.00 were considered positive. The demographic and laboratory data of all kidney transplant recipients were retrospectively reviewed from their medical records. Immunosuppressive regimens were divided into standard and low intensity regimen. Combination of tacrolimus (target C_trough_ 4–7 ng/mL) and mycophenolic acid represented the standard regimen. As for the low intensity regimens, it contained: (1) mTOR inhibitor (target C_trough_ 5–8 ng/mL) with low dose tacrolimus (target C_trough_ 2–4 ng/mL), (2) cyclosporine or azathioprine, or 3) immunosuppressive drugs other than CNI (CNI withdrawal regimen).

All eligible kidney transplant recipients with negative anti-HAV were randomized using the computer into 2 groups with a 2: 1 allocation ratio to receive either 2-dose (standard group) or 3-dose (study group) of inactivated hepatitis A vaccine [Havrix, GlaxoSmithKline plc (GSK) group of companies, London, United Kingdom] intramuscularly at 0, 6 and 0, 1, 6 months, respectively.

### Outcomes

The primary outcome was the seroconversion rate of anti-HAV IgG at 1 month after the last dose of hepatitis A vaccination. The secondary outcomes were to evaluate factors associated with seroconversion and to determine the seroepidemiology of HAV in kidney transplant recipients in Thailand. Safety outcomes were recorded during interviews at the outpatient clinic 3 days following each vaccination. Adverse events were collected and monitored.

### Statistical analysis

Based on a previous study conducted in South Korea, the anti-HAV seroconversion rate was 20% after standard vaccination. The sample size was calculated with 80% power and a two-sided type 1 error of 0.05^17^. A ratio of 2:1 was used to maximize the number of patients participating in a limited resource setting. The minimum required sample size was 29 for the three-dose group and 58 for the two-dose group.

Categorical and continuous variables were analyzed by Chi-squared and student T tests, respectively. Most numerical values with normal distribution were expressed as the mean and standard deviation. In multivariate analysis, a backward stepwise binary logistic regression with adjustment for covariates was used. All statistical analyses were performed utilizing the SPSS statistical analysis package (version 18.0.0; SPSS Inc., Chicago, Illinois, USA), and a *p* value of < 0.05 was considered statistically significant.

## Results

### Baseline seroprevalence of anti-HAV IgG antibody in kidney transplant recipients

A total of 401 kidney transplant recipients were eligible for the study and screened for HAV serology during outpatient clinic visit. The mean age of the participants was 49.9 ± 12.7 (range 18–78) years (Table 1). There were 191 (47.6%) living donor kidney transplant recipients and 235 (58.6%) participants were male. Among 401 kidney transplant recipients, 285 had positive anti-HAV IgG so the seroprevalence was 71.1%. Seropositive recipients were significantly older (age 54.3 ± 10.9 vs. 38.9 ± 9.9 years), male (62.1% vs. 50.0%), and had a longer time post-transplantation (98.9 ± 68.8 vs. 78.9 ± 57.7 months) compared to the seronegative recipients.

**Table 1:** Baseline characteristics of eligible kidney transplant recipients.

	Kidney transplant recipients (n = 401)	Seropositive (n = 285)	Seronegative (n = 116)	*p* value
Age (years, mean ± SD)	49.9 ± 12.7	54.3 ± 10.9	38.9 ± 9.9	< 0.001
Male gender (n, (%))	235 (58.6%)	177 (62.1%)	58 (50.0%)	0.026
Type of transplantation (living donors, n (%))	191 (47.6%)	139 (48.7%)	52 (44.8%)	0.473
Time post transplantation (months, mean ± SD)	93.2 ± 66.4	98.9 ± 68.8	78.9 ± 57.7	0.003
Serum creatinine (mg/dL, mean ± SD)	1.48 ± 0.98	1.46 ± 0.83	1.52 ± 1.28	0.588

### Post-vaccination seroconversion rate

**Figure 1:**
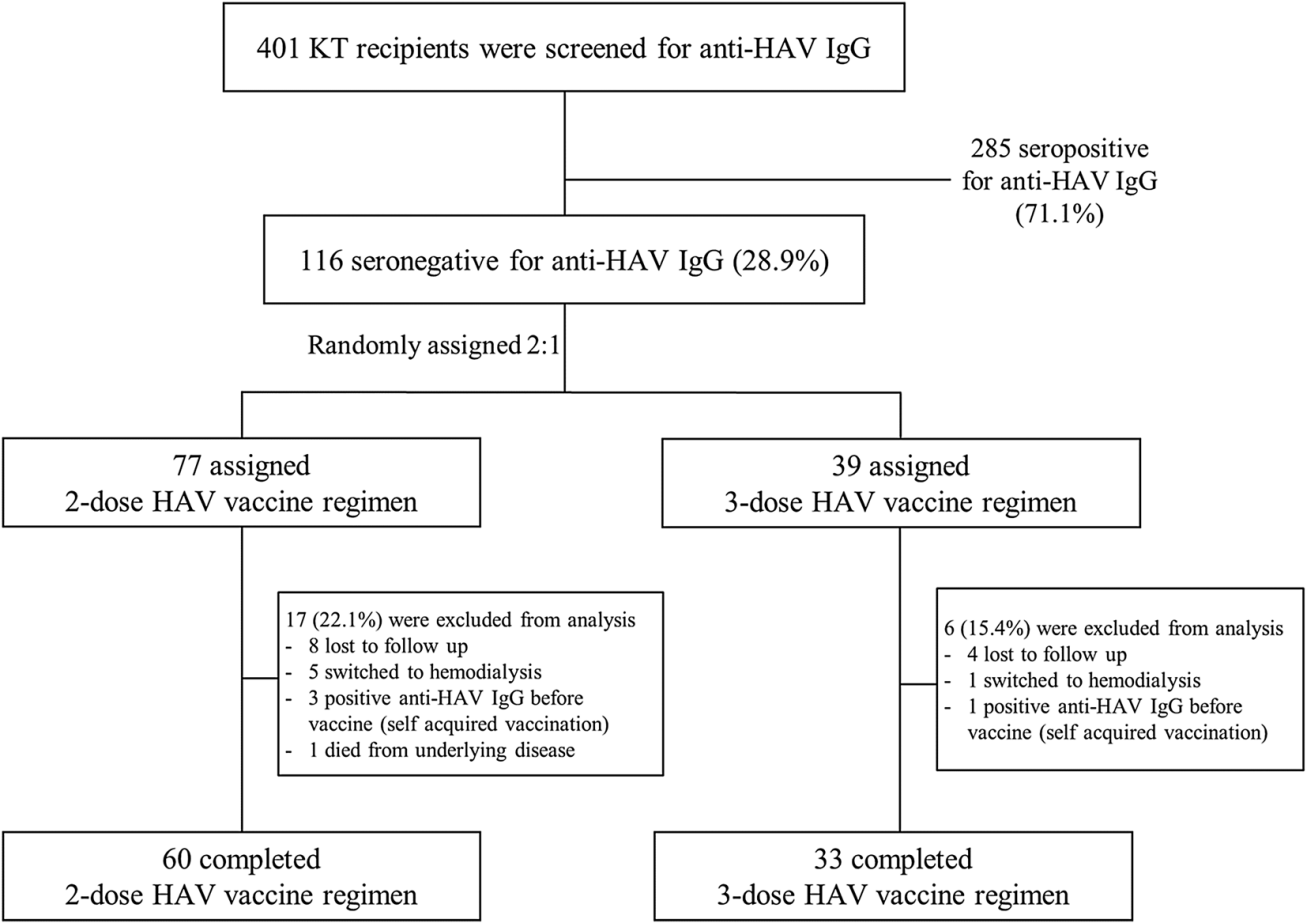
Enrollment and outcomes.

Of 116 kidney transplant recipients with negative anti-HAV IgG, 77 (66.4%) and 39 (33.6%) were assigned into the 2- and 3-dose groups, respectively (Fig. 1). Of 77 kidney transplant recipients in the 2-dose group, 8 (10.3%) were lost to follow-up, 5 (6.5%) had switched to chronic hemodialysis due to allograft failure, 3 (3.9%) had already been vaccinated with hepatitis A vaccine before enrollment, and 1 (1.3%) died from other underlying diseases. Of 39 kidney transplant recipients in the 3-dose group, 4 (10.3%) were lost to follow-up, 1 (2.6%) had switched to chronic hemodialysis due to allograft failure, and 1 (2.6%) had already been vaccinated with hepatitis A vaccine before enrollment. Therefore, 60 (77.9%) and 33 (84.6%) recipients in the 2- and 3-dose groups, respectively, had completed vaccination regimens (Fig. 1).

The baseline characteristics were not different between the 2 groups (Table 2). Among 93 kidney transplant recipients who had completed vaccination, seroconversion was observed in 47 participants (50.5%). The seroconversion rates after completing vaccination in the 2-dose and 3-dose regimens were 51.7% and 48.5%, respectively (p = 0.769) (Table 3).

**Table 2:** Baseline characteristics of the participants who received 2- and 3-dose regimens of hepatitis A vaccine.

	Total (n = 93)	2 doses (n = 60)	3 doses (n = 33)	*p* value
Age (years, mean ± SD)	38.1 ± 10.0	39.4 ± 10.4	35.9 ± 9.1	0.115
**Type of transplantation**				0.219
Living donor kidney transplant	40 (43%)	23 (38.3%)	17 (51.5%)	
Deceased donor kidney transplant	53 (57%)	37 (61.7%)	16 (48.5%)	
Time post transplantation (months, mean ± SD)	72.70 ± 51.98	78.98 ± 54.78	61.27 ± 45.03	0.116
**Immunosuppressive regimen**				0.195
Tacrolimus + mycophenolic acid	56 (60.2%)	34 (56.7%)	22 (66.7%)	
Tacrolimus minimization with mTOR inhibitor	14 (15.1%)	9 (15.0%)	5 (15.2%)	
Cyclosporine	12 (12.9%)	11 (18.3%)	1 (3%)	
Calcineurin inhibitor withdrawal	11 (11.8%)	6 (10.0%)	5 (15.2%)	
Prednisolone dose (Mean ± SD)	4.26 ± 4.86	4.06 ± 4.52	4.62 ± 5.48	0.598
Creatinine (mg/dL ± SD)	1.38 ± 0.63	1.39 ± 0.70	1.37 ± 0.48	0.906
eGFR (mL/min/1.73 m^2^)	64.82 ± 21.90	64.22 ± 22.92	65.91 ± 20.23	0.725
Hemoglobin (g/dL, mean ± SD)	12.87 ± 1.93	12.73 ± 1.85	13.12 ± 2.05	0.352
White blood cells count (cells/µL, mean ± SD)	7,159.71 ± 1,932.32	7,284.17 ± 2,001.55	6,930.61 ± 1,806.98	0.401
Percent lymphocyte (%)	29.37 ± 9.87	28.76 ± 10.28	30.48 ± 9.11	0.424
Lymphocyte count (cells/µL, mean ± SD)	2,045.02 ± 744.40	2,026.91 ± 778.05	2,077.93 ± 689.39	0.754
Albumin (g/dL, mean ± SD)	4.21 ± 0.32	4.20 ± 0.29	4.22 ± 0.37	0.748

eGFR; estimated glomerular filtration rate by CKD-EPI.

**Table 3:** Comparison of the vaccine response between 2- and 3-dose regimens.

	Total (n = 93)	2 doses (n = 60)	3 doses (n = 33)	*p* value
**Anti-HAV IgG after vaccination**				0.769
Negative (no seroconversion)	46 (49.5%)	29 (48.3%)	17 (51.5%)	
Positive (seroconversion)	47 (50.5%)	31 (51.7%)	16 (48.5%)	
Post titer (Mean ± SD)	4.18 ± 4.99	4.18 ± 4.91	4.16 ± 5.21	0.982

From the multivariate logistic regression analysis, three factors were significantly associated with seroconversion in both the two-dose and three-dose groups (Table 4). Participants with high estimated glomerular filtration rate (eGFR), high serum albumin level, and on low intensity immunosuppressive regimens had higher overall seroconversion rate (Table 4).

**Table 4:** Univariate and multivariate analysis of factors associated with seroconversion of hepatitis A vaccination in both groups (n = 93).

Factors	Univariate analysis		Multivariate analysis	
	OR (95%CI)	*p* value	OR (95%CI)	*p* value
Age (years)	1.004 (0.964–1.046)	0.853	0.991 (0.941–1.044)	0.734
**Gender**				
Male	1.042 (0.461–2.351)	0.922	0.919 (0.311–2.713)	0.905
Female	1.00 (reference)			
**Type of transplantation**				
Living donor kidney transplant	1.635 (0.714–3.741)	0.245	1.749 (0.576–5.313)	0.324
Deceased donor kidney transplant	1.00 (reference)			
Time post transplantation (months)	1.004 (0.996–1.012)	0.315	1.007 (0.993–1.020)	0.317
eGFR (1 mL/min/1.73 m^2^)	1.033 (1.011–1.056)	0.033	1.033 (1.005–1.063)	0.021
Hemoglobin	1.204 (0.963–1.507)	0.104	1.072 (0.783–1.468)	0.663
White blood cells count (cells/µL)	1.000 (1.000–1.000)	0.911	1.000 (1.000–1.000)	0.799
Lymphocyte count (cells/ µL)	1.000 (1.000–1.001)	0.263	1.000 (0.999–1.001)	0.975
Albumin (g/dL)	4.953 (1.235–19.872)	0.024	10.601 (1.557–72.164)	0.016
**Immunosuppressive regimen**				
Tacrolimus + mycophenolic acid	1.00 (reference)			
Low intensity regimens	3.939 (1.620–9.580)	0.002	4.552 (1.310–15.816)	0.017
Prednisolone (mg/day)	0.892 (0.782–1.018)	0.89	0.972 (0.850–1.111)	0.677
**Vaccine dose**				
2-dose	1.00 (reference)			
3-dose	1.136 (0.485–2.657)	0.769	0.944 (0.331–2.693)	0.914

eGFR; estimated glomerular filtration rate by CKD-EPI.

### Adverse events

Hepatitis A vaccination was well tolerated by all kidney transplant recipients. The adverse events were not statistically different between the two groups. Pain at injection site and localized myalgia occurred in some participants. No serious adverse events were observed among the participants in both groups. Hepatitis A vaccination had no effect on both the kidney allograft and liver function in any of the participants.

## Discussion

The results in the present study demonstrated that 285 of 401 kidney transplant recipients had positive results. The seroprevalence was 71.1%. Among seronegative recipients, the seroconversion rates at 1 month after vaccination in the standard 2-dose regimen (n = 60) and the 3-dose regimen (n = 33) were not different (p = 0.769) (Table 3). The adverse events were comparable between the two groups and were corrected by supportive treatment. Hepatitis A vaccination did not alter graft or liver function in kidney transplant recipients.

In a previous study, the overall seroprevalence for HAV in healthy Thai population was 48.6%^4^. In the present study, the seroprevalence was 71.1% among kidney transplant recipients which was comparable to the seroprevalence of Thais aged 41 to 50 years (75.8%)^4^. Since the mean age of our participants was 49.9 years, the seroprevalence of HAV in kidney transplant recipients in the present work was considered similar to the general population^4^. Besides the age which is a well-known factor, we further identified that the male gender and time post-transplantation were also associated with positive anti-HAV IgG (Table 1). The lower immunosuppression may contribute to higher positivity rate of HAV IgG later in the course of kidney transplantation.

As stated earlier, the epidemiology of HAV has changed. Now, there are higher rates of HAV and the severity of HAV infection in adult population and immunocompromised patients are higher compared to children^10^. In recent years, there were certain outbreaks of HAV infection in several parts of the world including United States and European countries^10,20–22^. This would underscore the crucial role of hepatitis A vaccination in at risk population.

Currently, hepatitis A vaccination is recommended for seronegative SOT recipients who live in or travel to endemic regions^13,14^. The seroconversion rates were 94 -100% after completing the 2-dose vaccination regimen at 0 and 6 months in the healthy population^12,23^. In contrast, immunogenicity among transplant recipients was less favorable. Therefore, immunocompromised patients remain at risk for HAV infection despite complete vaccination^24^. According to previous studies, the seroconversion rate of standard 2-dose regimen was 26.9–71.8% among kidney transplant recipients whereas for liver transplant recipients, the seroconversion rate was 26.1–97.4%^15,17,25^. It is unclear why there is a huge variation in the seroconversion rates between studies. It is possible that these differences may be due to the immunosuppressive regimens used and its intensity in suppressing the immune system. Moreover, the seroprevalence in each geographic area may also affect the seroconversion rate after hepatitis A vaccination. In a previous study, 18% and 29% of the patients with positive pre-transplant anti-HAV had lost their immunity 1 and 2 years after transplant, respectively^16^. Therefore, post-transplant vaccination probably acts as a booster dose and yields a higher chance of seroconversion among these patients^26^.

It has been widely documented that vaccinations for viral infections such as influenza or hepatitis B also had decreased immunological responses in SOT recipients^27^. Impairment of the cellular- and humoral-mediated immune responses from immunosuppressive agents are likely to compromise vaccine efficacy. Several strategies, such as higher dose, booster dose or adjuvanted vaccine, have been studied to improve immunogenicity of the influenza vaccine^28^. In addition, double-dose hepatitis B vaccination showed higher seroprotection rate among chronic kidney disease patients on hemodialysis^29^. For hepatitis A vaccine, the efficacy of the 3-dose regimen (additional dose of vaccine at week 4) had a higher seroconversion rate and geometric mean concentration of anti-HAV IgG among HIV patients^18,19^. Therefore, this seems to be an attractive strategy which can be implemented in SOT recipients.

In the present study, the effectiveness of the 3-dose regimen of hepatitis A vaccination was not superior to the standard 2-dose regimen among SOT recipients (Table 3). The seroconversion rates and the antibody titers were comparable between the two regimens. Hence, other strategies, including higher dose or adjuvated vaccine, should be further investigated. From multivariate analysis, only eGFR, serum albumin level and intensity of immunosuppressive regimen were associated with seroconversion rate of anti-HAV IgG. There was a positive correlation between renal allograft function and seroconversion rate among kidney transplant recipients receiving hepatitis A vaccine as well as chronic kidney disease patients receiving hepatitis B vaccine^17,30^. In addition, serum albumin level appeared to be an indicator of immunogenicity after hepatitis B vaccination among dialysis patients^31^. However, the roles of these two factors, renal allograft function and serum albumin, in immunological response remain unclear. On the other hand, the intensity of immunosuppression is likely to be an important factor in determining vaccine response in SOT recipients.

There were only two previous hepatitis A vaccination studies that determined the effectiveness of the standard 2-dose regimen on the seroconversion rate among kidney transplant recipients^15,17^. The comparative details of the two earlier works and the present study are shown in Table 5. The role of intensity of immunosuppression on seroconversion rate could be delineated when one examines the details of immunosuppressive regimen used in each study. Jeon et al^17^ and the present study showed that kidney transplant recipients on tacrolimus-containing regimen, which is considered as high intensity, had significantly lower seroconversion rate after hepatitis A vaccination. On the contrary, Stark et al. demonstrated that the seroconversion rate of anti-HAV IgG was up to 71.8% in kidney transplant recipients. Cyclosporine and azathioprine were the main immunosuppressive agents used while tacrolimus was used in minority of the patients^15^. This might explain why the seroconversion rate in Stark et al.’s study was higher than our results (Table 5).

**Table 5:** Comparison of hepatitis A vaccination studies conducted in kidney transplant recipients.

Author	Participant, n	Age (years)	Time post-transplant (months)	eGFR (mL/min/1.73 m^2^)	Albumin (g/dL)	Immunosuppression	**Seroconversion rate**
Stark et al.	N = 39	42.5 ± 11.4	Median 96 (IQR 78–139)	NA	NA	41% CsA/AZA/Pred 27% AZA/Pred 15% CsA/Pred	71.8%
Jeon et al.	N = 52	34.1 (range, 20–56)	Mean 60.78	59.39	4.4	76.9% Tac/MPA 23.1% CsA/MPA	26.9%
The present study	N = 93 (2-dose, n = 60 and 3-dose, n = 33)	38.1 ± 10.0	Mean 72.7 ± 51.98	64.82 ± 21.9	4.2 ± 0.32	60.2% Tac/MPA 15.1% mTORi/Tac minimization 12.9% CsA-based 11.8% CNI withdrawal	50.5% (2-dose, 51.7% and 3-dose, 48.5%)

IQR, interquartile range; CsA, cyclosporine A; AZA, azathioprine; Pred, prednisolone; eGFR, estimated glomerular filtration rate; Tac, tacrolimus; mTORi, mammalian target of rapamycin inhibitor; MPA, mycophenolic acid; CNI, calcineurin inhibitor.

There are certain strengths in the present study. Our study is the first, prospective, randomized controlled trial that measured the immunogenicity after standard 2-dose and 3-dose hepatitis A vaccination regimens were administered to kidney transplant recipients. The sample size in our study was more than the previous studies (Table 5). As with any study, there were some limitations. First, pre-transplant anti-HAV IgG was not tested. Thus, we were unable to identify the participants who had lost immunity after transplantation. It is possible that these people may have different seroconversion rate after hepatitis A vaccination. Last, the follow-up period after vaccination was short. A longitudinal study is needed to evaluate the immune durability between these 2 regimens.

In summary, seroconversion rate after hepatitis A vaccination in kidney transplant recipients was less favorable than the healthy population. Three-dose regimen did not show superior benefit over the standard 2-dose regimen. Other strategies such as pre-transplant vaccination, booster dose administration, double-dose vaccination or post-exposure immunoglobulin may be considered for kidney transplant recipients. Further studies regarding this issue are urgently needed.
